# First report of the Gondwana genus *Beatogordius* from India, with further records of two *Chordodes* species (Gordiida, Nematomorpha)

**DOI:** 10.3897/zookeys.643.10506

**Published:** 2017-01-05

**Authors:** Arun K. Yadav, Andreas Schmidt-Rhaesa, Limatemjen

**Affiliations:** 1 Department of Zoology, North-Eastern Hill University, Shillong 793 022, India; 2 Zoological Museum, University Hamburg, Martin-Luther-King-Platz 3, 20146 Hamburg, Germany; 3 Department of Zoology, Kohima Science College (Autonomous), Jotsoma, Nagaland 797002, India

**Keywords:** Beatogordius, biodiversity, Chordodes, Gordiida, Nematomorpha, new species

## Abstract

Three horsehair worms (Nematomorpha) are recorded from Nagaland, India. The first species is *Chordodes combiareolatus*, which was recently described from Nagaland. The second is Chordodes cf. furnessi, earlier reported from Meghalaya, and some new observations are added. The third is a new species, described as *Beatogordius nagalandis* **sp. n.** This is the first report of the genus *Beatogordius* in India and this observation fits well into the Gondwana distribution of the genus. The species is quite similar to *Beatogordius chinensis*, reported from South China.

## Introduction

Horsehair worms (Nematomorpha) are parasitic worms with a free-living phase in the life cycle. Thirteen species of horsehair worms (Nematomorpha: Gordiida) from five genera (*Gordius*, *Chordodes*, *Parachordodes*, *Paragordius*, *Gordionus*) have been reported from India until 1912 (see Schmidt-Rhaesa and Yadav 2004). Recently, this group of parasitic worms has achieved new attention in India, and since 2004 seven further species were newly described or newly recorded (Schmidt-Rhaesa and Yadav 2004, 2013, Schmidt-Rhaesa and Lalramliana 2011, 2016, Schmidt-Rhaesa et al. 2015). Six of these newly reported species belong to the genus *Chordodes*, and one to the genus *Acutogordius*, which had not been reported from India before.

In the present study a representative of the genus *Beatogordius* is described. This is the first species of this genus reported from India. Representatives of this genus are characterized by an arrangement of areoles, fine cuticular elevations, which are characteristic cuticular substructures in nematomorphs, arranged in longitudinal lines. The genus was erected by Heinze (1934) and has an interesting geographic distribution. All the 21 species currently known under the genus are mostly distributed in South America (De Villalobos et al. 2003), Africa (Schmidt-Rhaesa and de Villalobos 2002), and Madagascar (Schmidt-Rhaesa and Bryant 2004), with further reports from Australia (Schmidt-Rhaesa and Bryant 2004) and China (Schmidt-Rhaesa 2011). With the exception of China, the genus shows a perfect Gondwana distribution and the long time suspected occurrence in India is now confirmed.

In addition, new records are reported for the species *Chordodes combiareolatus* Schmidt-Rhaesa, Limatemjen & Yadav, 2015, and Chordodes cf. furnessi Montgomery, 1898, and supplement the previous documentations by new images.

## Material and methods

The specimens for the present study were collected by hand (for exact locations see below) and preserved in 70% ethanol. Pieces 2–3 mm long from the middle region of body, the anterior, and the posterior ends of body were processed for Scanning Electron Microscopy (SEM). The material was dehydrated in an increasing ethanol series, critically-point dried, and coated with gold in a sputter coater. Observations were made using LEO SEM 1524 at the University of Hamburg. Digital images of various body regions were taken.

## Results

### Beatogordius nagalandis sp. n.

Taxon classificationAnimaliaGordioideaChordodidae

http://zoobank.org/0522D719-7394-4017-9214-4A14F14C4F90

Figs 1 , 2

#### Type locality.

P-Khel Viswema Village, Kohima, Nagaland, India, from the community tap water. Collected by Mrs Bazule Toso.

#### Holotype.

A single male specimen from the type locality; alcohol preserved pieces of holotype deposited in the Museum of Department of Zoology, NEHU, Shillong with the accession no. MDOZ/NEHU/INV/112.

#### Host.

unknown

#### Etymology.

The species has been named after one of the Indian states, Nagaland, from where the specimen was collected.

#### Diagnosis.

Cuticle in midbody with longitudinal elevated ridges, which sometimes branch and fuse. Ridges highest in the central part, lower in lateral parts. Surface of ridges with fine cracks perpendicular to longitudinal axis. Cuticle between the ridges with 4 µm long spines. Ridges change to isolated areoles towards the anterior, anterior tip free of cuticular structures. Posterior end of male with two tail lobes, each lobe about three times as long as wide. Cloacal opening surrounded by spines, further spines in the region posterior of the cloacal opening. Anterolateral bristlefields are likely present. Adhesive warts with a keel are present on the ventral side anterior of the cloacal opening.

#### Description.

The holotype is 130 mm long, with a diameter of 0.37 mm in the midbody region. The specimen is very light (yellowish-white) in colour. The posterior end has two tail lobes.

**Figure 1.**
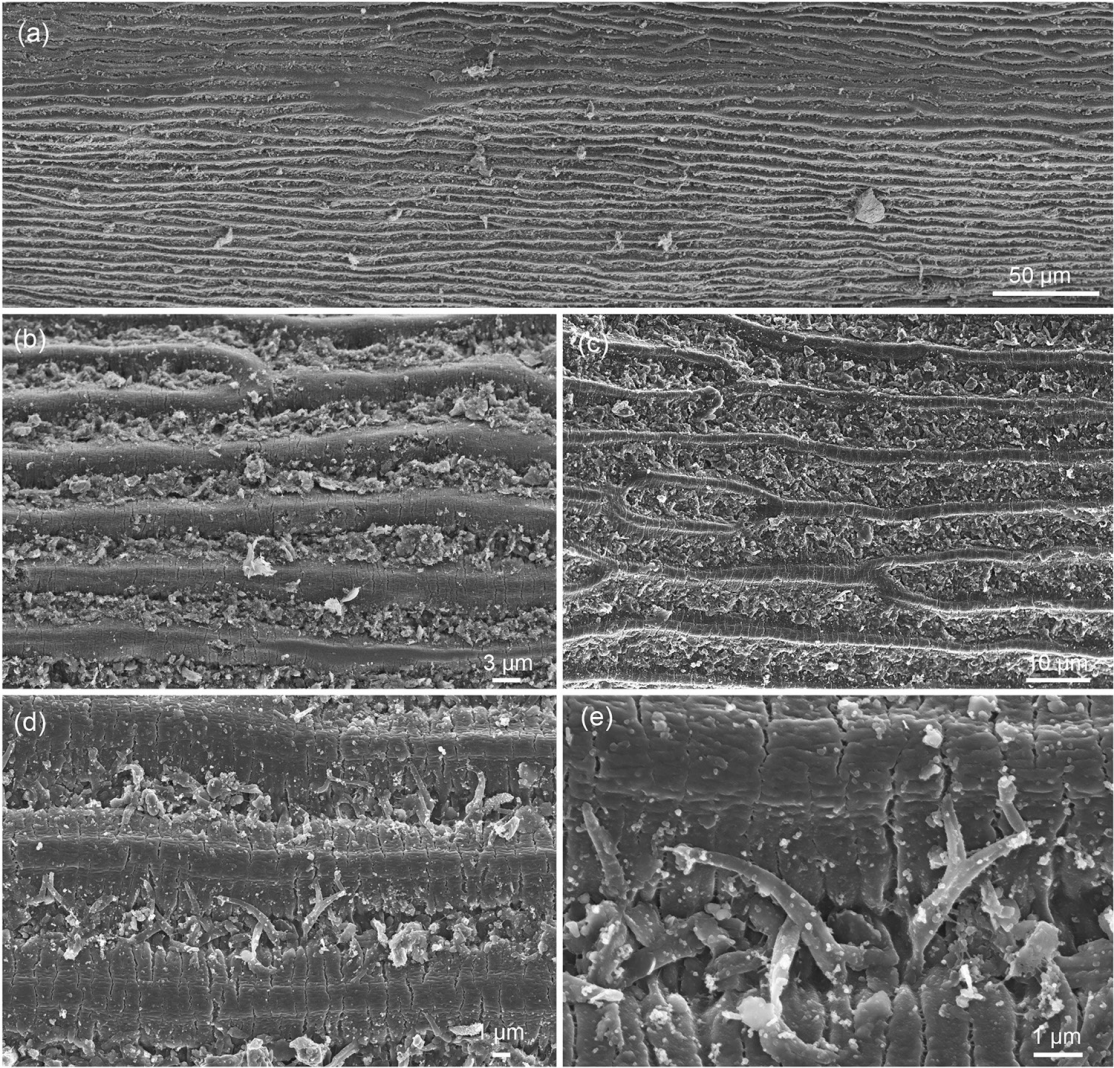
*Beatogordius nagalandis* sp. n., cuticular pattern: **a** cuticular pattern at lower magnification **b, c** branching and fusion of “bands”, space between bands is filled with debris **d, e** spines are present between the bands, fine cracks structure the bands stronger (d) or weaker (e) into subunits.

The cuticle is structured by long elevated bands or ridges running parallel to the longitudinal axis of the animal (Fig. 1a–d). These bands sometimes branch or fuse, and branches may end blindly (Fig. 1b, c). In most observed regions the bands are composed of a higher central part and lateral lower parts (Fig. 1d, e). In some regions the lower parts were not clearly observed, but this may be due to a covering of dirt in the region between the bands. The surface of the bands contains numerous fine cracks orientated perpendicular to their axis (Fig. 1d). In some regions these cracks appear without a certain pattern and are only partial (Fig. 1e), in others they appear to separate the band into numerous subunits (Fig. 1d). The region between the bands is often filled with debris, in parts where the dirt could be removed there are bristles or spines which are up to 4 µm long (Fig. 1d, e).

**Figure 2.**
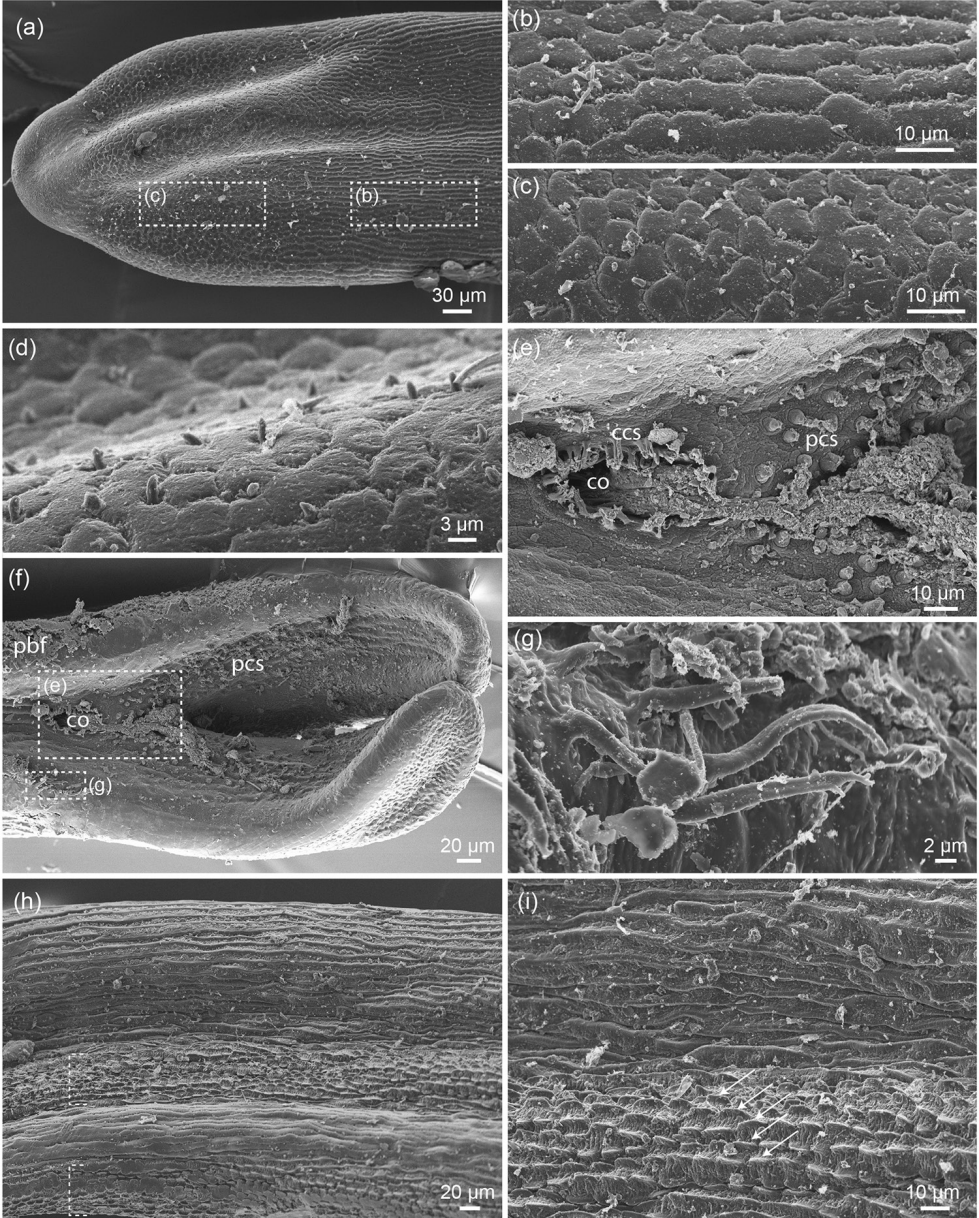
*Beatogordius nagalandis* sp. n., anterior and posterior end: **a** anterior end in low magnification, approximate positions of images b and c are indicated **b, c** transition of “bands” into areoles in two different regions as indicated in fig. a **d** fine spines are present between areoles in the anterior end **e** region of the cloacal opening (**co**) with circumcloacal spines (**ccs**) and postcloacal spines (pcs) **f** ventral view on posterior end with cloacal opening (**co**), postcloacal spines (**pcs**) and precloacal bristle fields (**pbf**). Approximate positions of images e and g are indicated by dotted rectangles **g** some bristles from the precloacal bristle fields **h** position of the broad rows of keeled structures (see dashed markings) on the ventral side of the animal **i** magnification of keeled structures (arrows).

In the anterior end, approximately 270 µm from the anterior tip, the longitudinal bands gradually turn into polygonal or irregularly shaped individual areoles (Fig. 2a–c). Between these areoles solid spines of 1–2 µm length are scattered (Fig. 2d). The anterior most 75 µm are free of cuticular structures (Fig. 2a).

The posterior end has two tail lobes and contains the ventral cloacal opening (Fig. 2f). This is oval, approximately 20 × 45 µm in size and surrounded by circumcloacal spines (Fig. 2e). These spines are stout, approximately 5 µm long, which are apically branched. The cloacal opening is 30–35 µm from the point where the separation into two tail lobes starts. The tail lobes are approximately three times as long as broad (roughly 300 × 100 mm). Posterior to the cloacal opening, extending onto the inner side of the tail lobes, are conical spines, which decrease in size posteriorly (Fig. 2e, f). Anterolaterally of the cloacal opening are structures which could represent parts of anterocloacal bristle fields, but as there is some dirt and suboptimal cuticle preservation in this region, this observation has to be taken with caution (Fig. 2f, g).

In the ventral region anterior of the posterior end, modifications of the cuticle were observed. There are paired stripes, in which elevation with longitudinal keels of 7.5 to 10 µm occur (Fig. 2h, i). Eight rows of such keeled structures occur in one stripe.

#### Taxonomic remarks.

In most *Beatogordius* species, areoles are clearly recognized as the basic elements, which form the longitudinal lines, and should be considered a characteristic of the genus (De Villalobos et al. 2003, Schmidt-Rhaesa and de Villalobos 2002). More or less continuous lines occur in *Beatogordius sankurensis* Sciacchitano, 1958 and *Beatogordius wilsoni* Sciacchitano, 1958 from Africa (see Schmidt-Rhaesa and de Villalobos 2002), *Beatogordius lineatus* Schmidt-Rhaesa & Bryant, 2004 from Australia (Schmidt-Rhaesa and Bryant 2004), and *Beatogordius chinensis* Schmidt-Rhaesa, 2011 from China (Schmidt-Rhaesa 2011). Among all the above-mentioned species, *Beatogordius chinensis* is the only species to display long spines in the region between the longitudinal bands. The difference from the Indian *Beatogordius* is that in *Beatogordius chinensis* the bands have a “pearl-collar” appearance, which means that they usually have broader and narrower regions, whereas in the Indian *Beatogordius* the bands are of continuous thickness. It might be supposed that the Indian and the Chinese species are closely related. *Beatogordius chinensis* is the only species reported outside former Gondwana continents, but the collecting locality in Yunnan province is very close to Nagaland province, approximately 300–400 km east.

### Chordodes combiareolatus

Taxon classificationAnimaliaGordioideaChordodidae

Schmidt-Rhaesa, Limatemjen & Yadav, 2015

Figure 3a–c

#### Locality.

Tzuden stream of Longjang village, Mokokchung district, Nagaland, India. Collected by Mr & Mrs Lanu Pongen.

#### Description.

**Figure 3.**
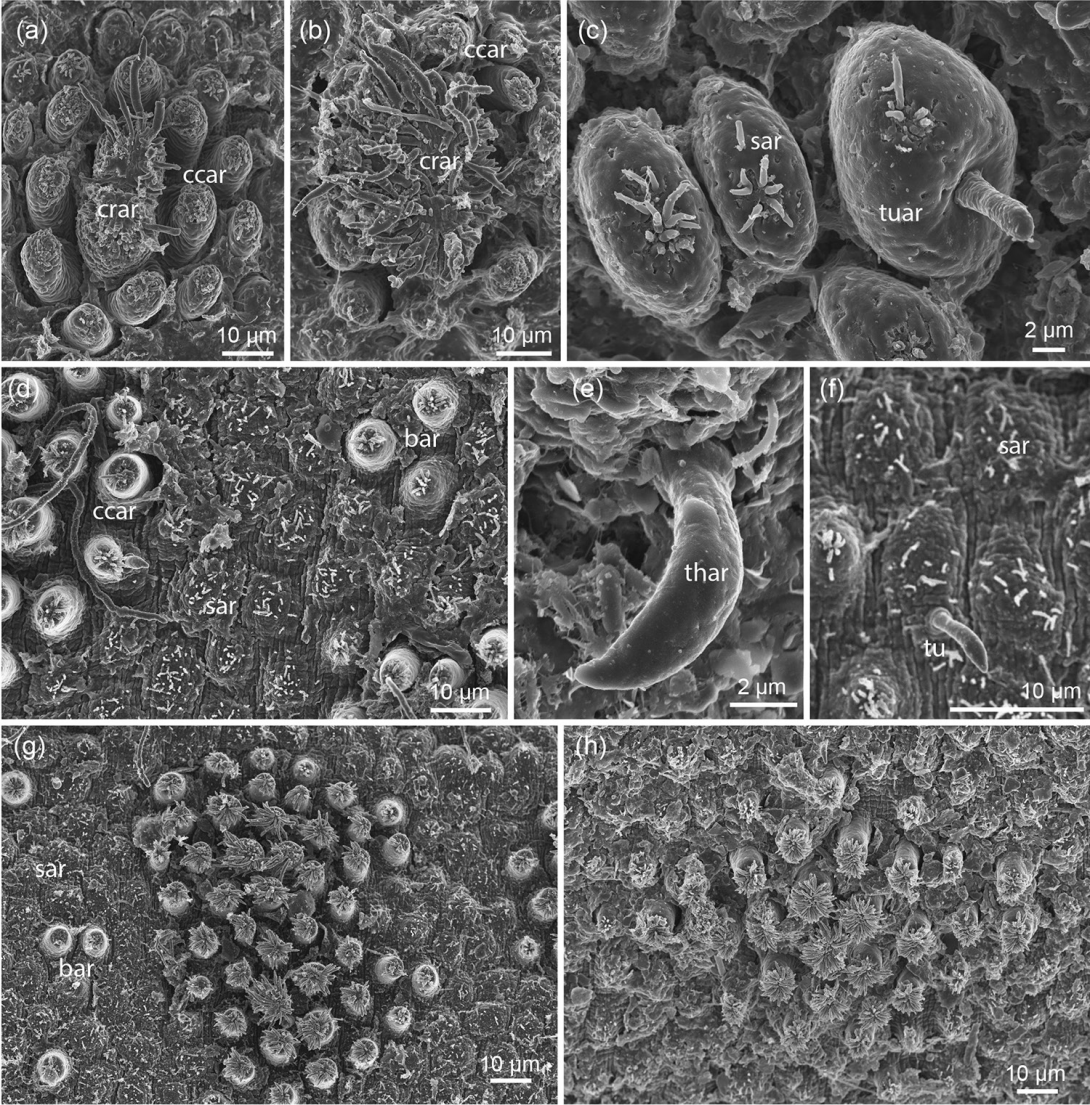
**a–c** *Chordodes combiareolatus*: **a, b** cluster of crowned areoles (**crar**) and circumcluster areoles (**ccar**) in different degrees of preservation of the apical filaments **c** simple areoles (**sar**) and the combined simple/tubercle areoles (**tar**) characteristic for this species **d–h** *Chordodes* cf. *furnessi*: **d** simple areoles (**sar**) and bulging areoles (**bar**) between clusters of crowned areoles (**ccar** = circumcluster areoles) **e** thorn areole (**thar**) **f** tubercle (**tu**) **g, h** two different clusters of crowned and circumcluster areoles, where no differences between these two types of areoles are visible.

Female specimen of 260 mm length and 1.5 mm in diameter. The cuticular characters correspond to the ones previously described by Schmidt-Rhaesa et al. (2015) for this species; the characteristic combination of simple and tubercular areoles are present (Fig. 3c). Crowned areoles were observed in different grades of completeness (Fig. 3a, b). The species had been described on the basis of a female specimen from Nagaland (Schmidt-Rhaesa et al. 2015); the male remains unknown.

### Chordodes cf. furnessi

Taxon classificationAnimaliaGordioideaChordodidae

(Montgomery, 1898)

Figs 3d–h , 4

#### Locality.

Tzuden stream of Longjang village, Mokokchung district, Nagaland, India. Collected by Mr & Mrs Lanu Pongen.

#### Description.

**Figure 4.**
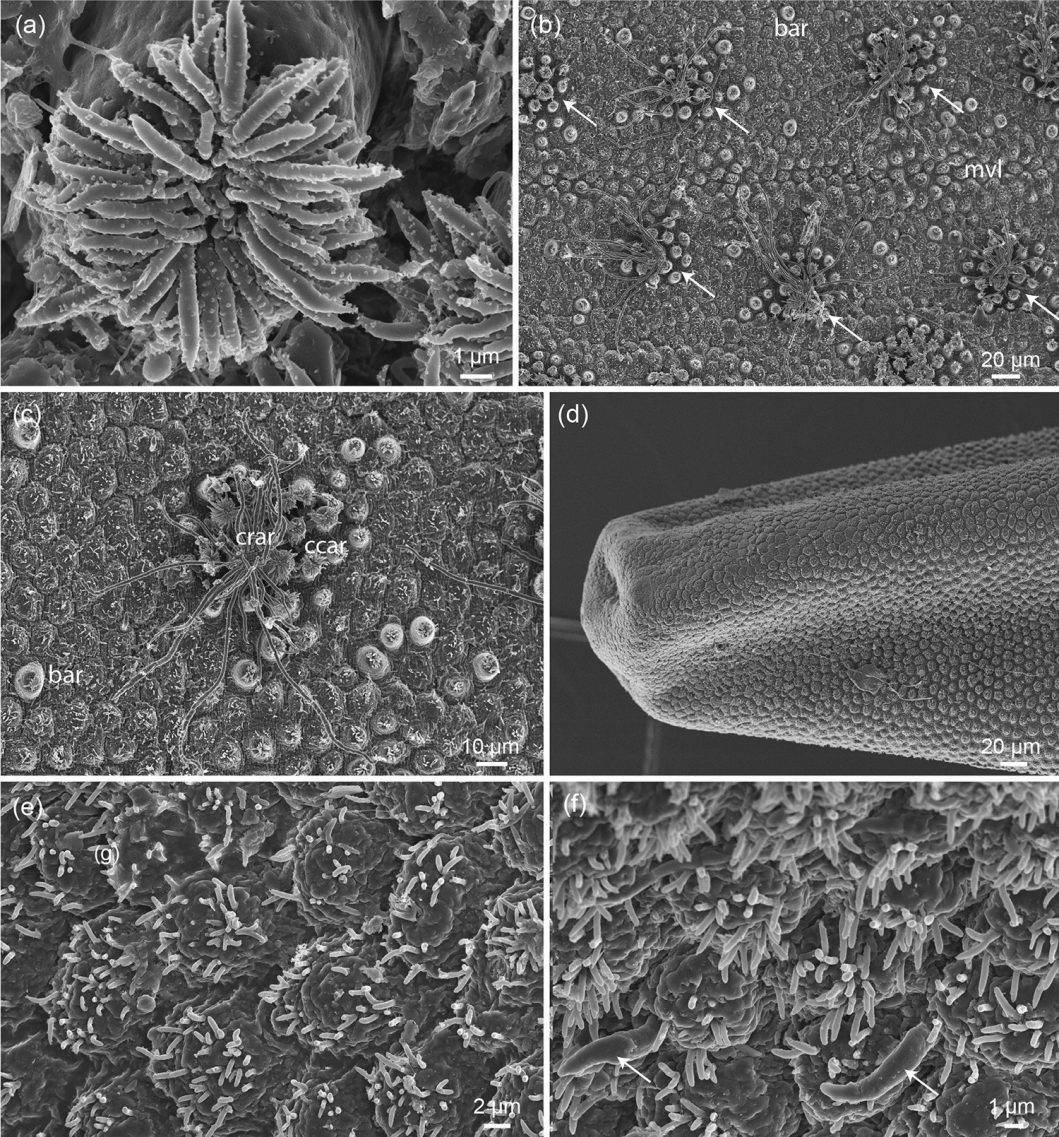
*Chordodes* cf. *furnessi*: **a** fine structure of areole from a crowned areole cluster **b** clusters containing crowned areoles with long apical bristles (arrowed) along the assumed midventral line (**mvl**) **c** in such clusters crowned areoles (**crar**) and circumcluster areoles (**ccar**) can be distinguished (bar = bulging areoles) **d** anterior end of the animal **e** fine structure of areoles in the anterior end **f** some spines (arrowed) are present between the areoles in the anterior end.

Female specimen of 264 mm length and 1.5 mm diameter. Simple areoles are flat and have fine bristles on top (Fig. 3d, f). Tubercle areoles and thorn areoles are present, but rare (Fig. 3e, f). Bulging areoles occur, mostly in pairs (Figs 3d, g, 4b, c). There is no distinction between crowned areoles and circumcluster areoles, all such areoles within one cluster have a similar shape and differ only slightly in size (Figs 3g, h, 4a). Crowned areoles with long apical filaments are present and restricted to a paired longitudinal line, presumably on the ventral side of the animal (Fig. 4b, c). Towards the anterior end there is a transition of the cuticular pattern; only one type of areole is present in the anterior end, which resembles simple areoles, is moderately elevated and has numerous bristles on top (Fig. 4d-f). Scattered spines are present between these areoles (Fig. 4f).

#### Taxonomic remarks.

In most *Chordodes* species, there are clusters composed of central crowned areoles and surrounding circumcluster areoles. These two types differ in structure but in some species, clusters are composed of areoles of the same type: *Chordodes furnessi* Montgomery, 1898 is one such species, first described from Borneo (Indonesia). Schmidt-Rhaesa and Yadav (2004) found specimens from Shillong, India which is very similar to this species, but, because of slight differences observed, it was recorded as *Chordodes* cf. *furnessi*. This newly reported specimen corresponds to the description given by Schmidt-Rhaesa and Yadav (2004), but adds some further observations. Bulging areoles were not previously reported, but they are figured (Fig. 1b in Schmidt-Rhaesa and Yadav 2004). Solid thorns have not been reported previously from this species, but are present in the newly reported specimen. Tubercles occur usually in the genus *Chordodes* as tubercle areoles, i.e., as areoles with a central tubercle on top, but in the present specimen, they occur near the base of simple areoles, as reported by Schmidt-Rhaesa and Yadav (2004). Due to these observations it is assumed that the specimens reported by Schmidt-Rhaesa and Yadav (2004) and in this work belong to the same species, which may or may not be *Chordodes furnessi*. The lack of rare structures such as thorns may be due to the scarceness of these structures.

## Supplementary Material

XML Treatment for Beatogordius nagalandis

XML Treatment for Chordodes combiareolatus

XML Treatment for Chordodes cf. furnessi
